# CircPTPRA blocks the recognition of RNA *N*^*6*^-methyladenosine through interacting with IGF2BP1 to suppress bladder cancer progression

**DOI:** 10.1186/s12943-021-01359-x

**Published:** 2021-04-14

**Authors:** Fei Xie, Chao Huang, Feng Liu, Hui Zhang, Xingyuan Xiao, Jiayin Sun, Xiaoping Zhang, Guosong Jiang

**Affiliations:** 1 Department of Urology, Union Hospital, Tongji Medical College, Huazhong University of Science and Technology, Wuhan, 430022 China; 2 Department of Urology, The Affiliated Hospital of Qingdao University, Qingdao, 266013 China

**Keywords:** Bladder cancer, circPTPRA, IGF2BP1, *N*^*6*^-methyladenosine

## Abstract

**Background:**

Circular RNAs (circRNAs) have been found to have significant impacts on bladder cancer (BC) progression through various mechanisms. In this study, we aimed to identify novel circRNAs that regulate the function of IGF2BP1, a key m^6^A reader, and explore the regulatory mechanisms and clinical significances in BC.

**Methods:**

Firstly, the clinical role of IGF2BP1 in BC was studied. Then, RNA immunoprecipitation sequencing (RIP-seq) analysis was performed to identify the circRNAs interacted with IGF2BP1 in BC cells. The overall biological roles of IGF2BP1 and the candidate circPTPRA were investigated in both BC cell lines and animal xenograft studies. Subsequently, we evaluated the regulation effects of circPTPRA on IGF2BP1 and screened out its target genes through RNA sequencing. Finally, we explored the underlying molecular mechanisms that circPTPRA might act as a blocker in recognition of m^6^A.

**Results:**

We demonstrated that IGF2BP1 was predominantly binded with circPTPRA in the cytoplasm in BC cells. Ectopic expression of circPTPRA abolished the promotion of cell proliferation, migration and invasion of BC cells induced by IGF2BP1. Importantly, circPTPRA downregulated IGF2BP1-regulation of MYC and FSCN1 expression via interacting with IGF2BP1. Moreover, the recognition of m^6^A-modified RNAs mediated by IGF2BP1 was partly disturbed by circPTPRA through its interaction with KH domains of IGF2BP1.

**Conclusions:**

This study identifies exonic circular circPTPRA as a new tumor suppressor that inhibits cancer progression through endogenous blocking the recognition of IGF2BP1 to m^6^A-modified RNAs, indicating that circPTPRA may serve as an exploitable therapeutic target for patients with BC.

**Supplementary Information:**

The online version contains supplementary material available at 10.1186/s12943-021-01359-x.

## Background

Bladder cancer (BC) is the most common urinary system malignancy with an estimated 81,400 new cases in 2019 in the United States [1]. For patients with advanced-stage or chemotherapy-refractory BC, the prognosis remains poor in despite of improvements in surgical techniques and medical therapy [2, 3]. The development of BC is a complex process and epigenetic abnormalities have been demonstrated to play critical roles in BC pathogenesis [4, 5], including DNA and histone modifications, chromatin remodeling, RNA methylation and so on. In particular, *N*^6^-methyladenosine (m^6^A), the most prevalent RNA methylation, is emerging as critical regulator in multiple fundamental biological processes [6, 7]. However, our understanding of the regulatory roles and the underlying mechanisms for m^6^A in BC is still limited.

Circular RNAs (circRNAs) are a novel class of single-stranded RNAs and characterized by covalently closed continuous loops and resistance to RNase R digestion [8]. Increasing RNA-sequencing analyses have revealed evolutionary conservation and abundance of circular RNAs, suggesting specific roles of circRNAs in cellular physiology [9, 10]. Specifically, circRNAs have been verified as “microRNA (miRNA) sponges,” harboring multiple miRNAs and functioning as miRNA inhibitors [11]. For example, our previous research demonstrated that BCRC-3 suppressed proliferation of BC cells through miR-182-5p/p27 axis [12]. Nevertheless, genome-wide studies have demonstrated that miRNA sponging activity cannot be generally applied, and other mechanisms have also been proposed, such as acting as platforms for protein interaction, translating into peptides or proteins [13, 14]. For example, overexpressed circSTAG1 captured ALKBH5 and decreased the translocation of ALKBH5 into the nucleus, leading to increased m^6^A methylation of fatty acid amide hydrolase (FAAH) messenger RNA [15]. Accumulating evidences show that circRNAs are frequently deregulated in various human cancers and participate in multiple biological processes [16]. However, the roles and mechanisms of circRNAs in the process of recognitions of m^6^A methylation remain largely elusive.

The insulin-like growth factor-2 mRNA-binding protein 1 (IGF2BP1), also known as IMP1, CRD-BP, ZBP1, or VICKZ1, belongs to a conserved family of RNA-binding, oncofetal proteins (IGF2BP1–3). Recent studies indicate that IGF2BP1 has the most conserved ‘oncogenic’ role of the IGF2BP family in tumor-derived cells, by affecting RNA stability, translatability, or localization [17]. Crosslinking immunoprecipitation (CLIP) analyses showed that IGF2BPs preferentially recognize m^6^A-modified mRNAs and facilitate the stability and translation of potential mRNA targets in an m^6^A-dependent manner, therefore having impacts on gene expression output [18, 19]. Recently, non-coding RNAs (ncRNAs) have been demonstrated to be involved in modulating the expression and function of IGF2BP1. For example, the post-transcriptional control of IGF2BP1 expression by let-7 microRNAs is suggested to modulate tumor cell fate [20]. A conserved direct interaction of the lncRNA THOR with IGF2BP1 showed that THOR contributes to the mRNA stabilization activities of IGF2BP1 [21]. However, it remains poorly understood the involvement of IGF2BP1 in BC development and how it might be modulated by circular RNA.

In this study, we revealed the oncogenic functions of IGF2BP1 in BC progression and identified a number of novel circular RNAs interacting with IGF2BP1 through high-throughput sequencing. A circRNA derived from PTPRA pre-mRNA (circPTPRA) was screened out. It showed that circPTPRA could suppress the growth and aggressiveness of BC cells by competitively binding with KH domains of IGF2BP1 and blocking its interaction with downstream target m^6^A-modified mRNA, MYC and FSCN1. The results of this study delineate novel mechanisms of circRNA/IGF2BP1-mediated regulation of tumor progression and provide opportunities for therapeutic intervention in BC.

## Methods

### Human tissue specimens

A total of 64 pairs of BC tissues and corresponding adjacent noncancerous bladder epithelial tissues were obtained from patients who underwent radical cystectomy in Union Hospital of Tongji Medical College of Huazhong University of Science and Technology (Wuhan, China), from 2015 to 2018. All the specimens were confirmed by at least two experienced histopathologists independently according to the criteria of the sixth edition TNM classification of the International Union Against Cancer. All specimens were snap-frozen in liquid nitrogen upon collection and stored at − 80 °C until use. Clinical information of the patients with BC was listed in Table S1. The study was approved by the Tongji Medical College of Huazhong University of Science and Technology Research Ethics Committee, and each patient signed informed consent before the research started.

### Cell culture

BC cell line (EJ) was purchased from American Type Culture Collection (ATCC, USA). The human metastatic bladder cancer cell line T24T, a lineage-related lung metastatic variant of invasive bladder cancer cell line T24, was obtained from the Departments of Urology, University of Virginia (Charlottesville, VA) as a gift in 2010 and was subjected to DNA tests and authenticated in our previous studies. T24T was cultured in DMEM (Invitrogen, USA) supplemented with 10% FBS (Gibco, USA) and 1% penicillin/streptomycin (Gibco, USA). EJ was cultured in RPMI1640 (Gibco, USA) supplemented with 10% FBS, 1% penicillin/streptomycin. All cell lines were confirmed 3 months before the beginning of the study based on a short tandem repeat method and were tested negative for mycoplasma contamination.

### RT–PCR and real-time quantitative RT–PCR

Total RNA was isolated from tissues and cell lines with RNeasy Mini kit (QIAGEN, Germany) according to the manufacturer’s instructions. RNA was reverse transcribed using the PrimeScript RT Master Mix (Takara, Japan). Real-time PCR was performed using SYBR Premix Ex TaqTM kit (Takara, Japan) and primers (Table S2). The results were analyzed with the Step OnePlus Real Time PCR System (Applied Biosystems, USA). The 2^-△△Ct^ method was used to quantify the transcript levels.

### CRISPR/Cas9 KO

T24T and EJ cells were transiently transfected with the lentiCRISPR v2 plasmid (Addgene Plasmid #52961) containing IGF2BP1 single-guide RNA (sgRNA) using Lipofectamine 2000 (Invitrogen) following the manufacturer’s instructions. Single-cell colonies were selected, and knockout efficiency was tested by Western blot analysis and Sanger sequencing. For Sanger sequencing, genomic DNA was extracted from sgCtrl and sgIGF2BP1#1 cells. PCR was performed to amplify the region flanked by the target site. The sgRNA sequences used are listed below: sgIGF2BP1#1, TATTCCACCCCAGCTCCGAT; sgIGF2BP1#2, GAGCGTGACCCCCGCGGACT.

### Western blot

Tissue or cellular protein was extracted with RIPA Lysis Buffer (Thermo Scientific, USA) according to the instructions. The concentration of total protein was measured by bicinchoninic acid (BC) protein assay kit (Beyotime, China). Western blot was conducted as previously described, with antibodies specific for IGF2BP1 (ab82968), MYC (ab32072), FSCN1 (ab126772), HSPA6 (ab69408), FLAG (ab1162), GAPDH (ab8245), and β-actin (ab8226).

### Immunohistochemistry

Immunohistochemistry was performed as previously described, with antibodies specific for Ki-67 (Proteintech, 1:200) or CD31 (Proteintech, 1:200). Images were captured by an Olympus FSX100 microscope (Olympus, Japan). Protein expression levels were analyzed by image-pro Plus 6.0. software through calculating the integrated optical density per stained area (IOD/area).

### In vivo growth and metastasis assays

All animal experiments were carried out in accordance with NIH Guidelines for the Care and Use of Laboratory Animals and approved by the Animal Care Committee of Tong ji Medical College (approval number: 20192290). For in vivo tumor growth studies, 1 × 10^6^ treated T24T cells were subcutaneously injected into the right axilla of blindly randomized four-week-old female BALB/c nude mice (*n* = 5 per group). Four weeks after injection, the mice were sacrificed. Tumor growth rates were monitored every other day, and tumor volume was calculated according to the formula (Tumor volume = π/6 × length×width^2^). For in vivo metastasis studies, 2 × 10^6^ treated T24T cells were injected into each blindly randomized 4-week-old BALB/c nude mice (*n* = 5 per group) through the tail vein. Ten weeks after injection, the mice were sacrificed. The survival time of each mouse was monitored and recorded. The In-Vivo FX PRO small animal imaging system (BRUKER Corporation, USA) was used to obtain fluorescence images of xenografts in nude mice.

### RNA fluorescence in situ hybridization

RNA Fluorescence in situ hybridization was performed according to the manufacturer’s instructions. Cy3-labeled circPTPRA probes were designed and synthesized by RiboBio (Guangzhou, China). The signals of circPTPRA were detected by Fluorescent In Situ Hybridization Kit (RiboBio, China). The images were captured using Nikon A1Si Laser Scanning Confocal Microscope (Nikon Instruments Inc., Japan).

### Fluorescence immunocytochemical staining

BC cells were grown on coverslips, and treated with antibodies specific for IGF2BP1 (8482S, CST; 1:100 dilution) at 4 °C overnight. Then, coverslips were treated with Alexa Fluor 488 goat anti-rabbit IgG (1:400 dilution) and DAPI (300 nmol/L) staining. The images were photographed under a Nikon A1Si Laser Scanning Confocal Microscope (Nikon Instruments Inc., Japan).

### Cell cycle assay

Cell cycle analysis was performed by flow cytometry. Cells were harvested and fixed in 75% ice-cold ethanol at 4 °C overnight. The fixed cells were washed with PBS twice and then stained with propidium iodide (PI) buffer (BD Pharmingen, USA). Then, cell cycle analysis was performed by FACS scan flow cytometer. ModFit LT 2.0 was used to analyze the data.

### In vitro cell migration and invasion assays

The abilities of cell migration and invasion were measured using transwell chambers (corning, USA) with 8 μm pore filters according to the manufacturer’s instructions. Cells were suspended in 200 μl serum-free medium (5 × 10^4^ cells per well for migration, and 1 × 10^5^ per well for invasion) and were added to the upper chambers coated with or without 50 μl of Matrigel (BD Biosciences, USA). DMEM medium containing 20% FBS was added to the bottom of chambers. After incubation at 37 °C for 24 h (migration assay) or 48 h (invasion assay), cells on the upper surface were removed with a cotton swab. Cells that migrated or invaded into the bottom of the membrane were fixed with 4% paraformaldehyde, stained with crystal violet solution, and then visualized under a microscope. The cell numbers were counted in five random fields of view.

### RNA pull-down assays

The biotinylated probe of circPTPRA was designed and synthesized by RiboBio (Guangzhou, China). The sequence of circPTPRA probe was listed in Table S2. Pull-down assay was performed as described in our previously studies. Briefly, M-280 streptavidin magnetic beads (Invitrogen, USA) at 25 °C for 2-4 h, and then total cell lysates with Protease/Phosphatase Inhibitor Cocktail and RNase inhibitor added were incubated with circPTPRA probe or oligo probe at 4 °C overnight. After washing thoroughly three times, the RNA complexes bound to the beads were eluted and extracted with RNeasy Mini Kit (QIAGEN) and were analyzed by qRT-PCR, and the RNA–protein binding mixture was boiled in SDS buffer and the eluted proteins were detected by western blot.

### RNA stability assay for mRNA lifetime

T24T cells with stably overexpressed circPTPRA were seeded into 6-well plates to get 50% confluency after 24 h. Cells were treated with 5 μg/ml actinomycin D and collected at indicated time points. The total RNA was extracted by miRNeasy Kit (Qiagen) and analyzed by RT-PCR. The turnover rate and half-life of mRNA was estimated according to previously published paper [18, 22]. The primers for MYC and FSCN1 are listed in Table. S2.

### RNA immunoprecipitation (RIP)

Cells seeded in a 15-cm dish at 70–80% confluency were cross-linked by ultraviolet light at 254 nm (200 J/cm^2^), then harvested and lysated. RNA immunoprecipitation (RIP) assay was performed according to the instructions of the Magna RIP RNA Binding Protein Immunoprecipitation Kit (Millipore, USA), using antibody specific for IGF2BP1 (8482S, CST), FLAG (ab1162, abcam) or a corresponding control IgG (mouse IgG (CS200621, Millipore) for FLAG, rabbit IgG (NI01, Millipore) for IGF2BP1. Input and co-immunoprecipitated RNAs were extracted with a RNeasy Mini kit (QIAGEN, Germany) according to the manufacturer’s instructions and analyzed by qRT-PCR or RNA-seq.

### RNA sequencing

Total RNA was isolated from circPTPRA-overexpressed T24T and EJ cells and the corresponding control cells using RNeasy Mini kit (Qiagen). Transcriptome sequencing on an Illumina HiSeq X Ten platform was carried out by SeqHealth Tech (Wuhan, China).

### Dual-luciferase reporter assay

DNA fragments of wild-type and mutant CRD were synthesized and cloned into the pMIR-REPORT vector (Promega, USA) to constructed CRD firefly luciferase reporters.

HEK293T cells were seeded in 24-well plates at 60–80% confluency before transfection. The CRD firefly luciferase reporter plasmids (pMIR-CRD-WT and pMIR-CRD-mut, respectively) and renilla luciferase reporter control vectors (pRL-TK) were co-transfected with circPTPRA plasmid or vectors to examine the CRD luciferase activities. On the other hand, the pMIR-CRD-wide type reporter plasmids were co-transfected with circPTPRA plasmids and IGF2BP1 expression plasmids using Lipofactamine 2000 (Invitrogen). The firefly and renilla luciferase activities were measured after 48 h with Dual-Luciferase® Reporter Assay System (Promega, USA) according to the manufacturer’s protocol.

### Statistical analysis

All the data statistical analyses were performed using GraphPad Prism 7.0 software (La Jolla, USA) to assess the differences between groups. Data were shown as mean ± SEM. The chi-squared test was used to assess the association of the expression of IGF2BP1 or circPTPRA with the patient’s clinicopathologic characteristics. Kaplan–Meier survival curve was employed to depict the OS distributions and Log-rank test was used to assess survival difference. Independent sample t test was employed to assess statistical significance of comparisons between groups. Pearson’s correlation coefficient assay was used to analyze the expression correlation. One-way analysis of variance was performed to evaluate the group difference. *P* < 0.05 was considered statistically significant.

## Results

### IGF2BP1 was up-regulated in BC and could promote BC cells invasion, metastasis and cell cycle progression in vitro and in vivo

**Fig. 1:**
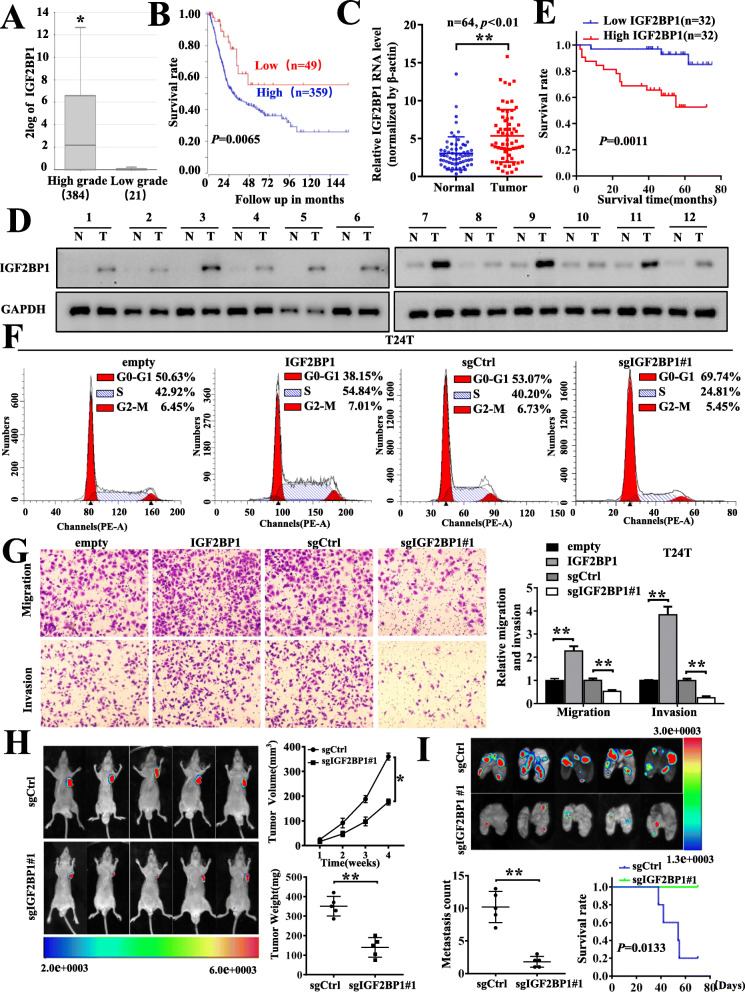
IGF2BP1 was up-regulated in BC and could promote BC cells progression in vitro and in vivo. **a** The mRNA expression levels of IGF2BP1 in BC obtained from TCGA datasets. **b** The Kaplan–Meier curves with univariate analyses of overall survival (OS) in BC patients with low versus high expression of IGF2BP1 from TCGA cohorts. **c** The expression of IGF2BP1 in BC tissues (Tumor) compared to paired adjacent normal bladder tissues (Normal) of 64 clinical patients. **d** Western blot indicating IGF2BP1 protein levels were significantly upregulated in BC tissues compared with adjacent normal bladder epithelial tissues. **e** Kaplan–Meier’s analyze of correlation between IGF2BP1 expression level and overall survival of 64 patients with BC (the patients were divided into high- and low-expression groups using the median value as the cut-off point, *n* = 64, *p* = 0.0011, log-rank test). **f** and **g** Cell cycle analysis (**f**) and representative images (left) and quantification (right) of transwell assay (**g**) indicating the proliferation and invasion of T24T cells with stable overexpression or knockout of IGF2BP1. **h** Representative images, in vivo growth curve (bottom right, up), and weight at the end points (bottom right, down) of xenografts formed by subcutaneous injection of T24T cells transfected with CRISPR-vector, or CRISPR Cas9-IGF2BP1 into the dorsa flanks of nude mice (*n* = 5 for each group). **i** Representative images, and quantification (bottom left) of lung metastatic colonization and Kaplan–Meier curves (bottom right) of nude mice treated with tail-vein injection of T24T cells stably transfected with sgCtrl or sgIGF2BP1#1 (*n* = 5 for each group). *, *P* < 0.05; **, *P* < 0.01

To investigate the clinical role of IGF2BP1 in BC, we first determined IGF2BP1 expression in The Cancer Genome Atlas (TCGA) RNA-seq dataset and revealed that IGF2BP1 mRNA expression was significantly higher in high grade tumor tissues (*n* = 384), compared with low grade tumor tissues (*n* = 21) (**P < 0.001) (Fig. 1a). Moreover, Kaplan–Meier survival curves demonstrated that high level of IGF2BP1 was significantly associated with poor overall survival (OS) (Fig. 1b). Next, we compared the expression of IGF2BP1 in tumor tissues compared to that in matched normal tissues derived from 64 BC patients in our single-center. Consistent with those identified in TCGA dataset, IGF2BP1 mRNAs were significantly up-regulated in human BC tissues, compared with those in adjacent noncancerous tissues (Fig. 1c). Consistently, the elevated level of IGF2BP1 protein was noted in 12 pairs of tumor tissues than those of adjacent non-cancerous tissues (Fig. 1d). Furthermore, the relationship between IGF2BP1 mRNA levels and the clinical pathological features were analyzed in the 64 BC and adjacent non-cancerous tissues. The results showed that the expression of IGF2BP1 was positively correlated with tumor size, the metastasis of lymph nodes, and advanced clinical stages of BC, respectively (Table. S1). Importantly, high expression level of IGF2BP1 was remarkably associated with poor prognosis of BC patients (Fig. 1e).

To further explore the oncogenic properties of IGF2BP1 in BC, we established stable models of IGF2BP1 overexpression or knockout in EJ and T24T cell lines, respectively (Fig. S1A, B). The knockout efficiency was tested by Western blot analysis and Sanger sequencing (Fig. S1B, C). Stable overexpression or knockout of IGF2BP1 strikingly facilitated or reduced the cell cycle progression, proliferation, migration and invasion of BC cells in vitro, respectively (Fig. 1f, g& Fig. S1D, E, F). Consistently, knockout of IGF2BP1 in T24T cells led to a significant decrease in growth and tumor weight of subcutaneous xenograft tumors (Fig. 1h), and less lung metastatic counts and more survival of nude mice (Fig. 1i). Together, these findings firstly determined the oncogenic roles of IGF2BP1 in BC growth and aggressiveness in vivo and in vitro.

### IGF2BP1 could interact with circPTPRA in the cytoplasm in BC cells

**Fig. 2:**
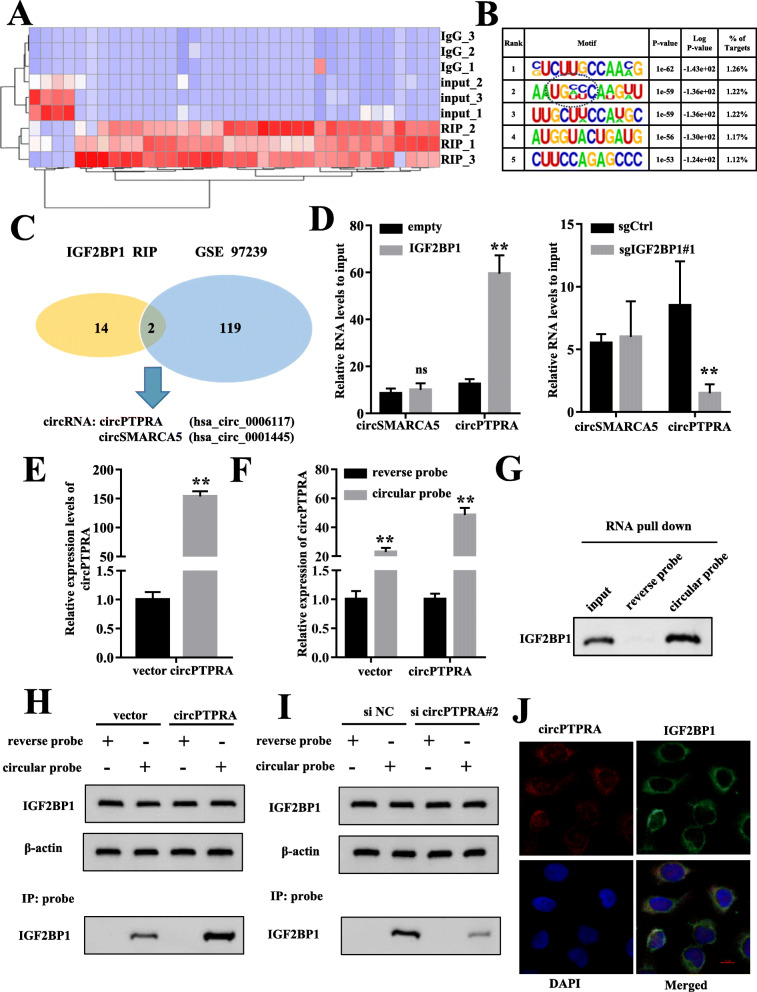
IGF2BP1 could interact with circPTPRA in the cytoplasm in BC cells. **a** Heatmap of the RIP-seq experiments showing the IGF2BP1-binding circRNAs in bladder cancer (BC). **b** Top consensus sequences of IGF2BP1-binding sites and the m^6^A motif detected by HOMER motif analysis. **c** Overlap of IGF2BP1-binding circRNAs identified by RIP–seq and published GSE 97239 dataset in BC. **d** RIP and qRT-PCR assays using an antibody specific for IGF2BP1 showing the interaction between circRNAs (circPTPRA, circSMARCA5) and IGF2BP1 in T24T cells with stable overexpression or knockout of IGF2BP1. **e** qRT-PCR analysis verified the efficiency of circPTPRA overexpression and pulled down by biotin-labeled reverse or circular RNA probes from lysates of T24T cells. **f** Cell lysates prepared from T24T cells transfected with circPTPRA or the vector were hybridized with circular probe or reverse probe for RNA pull-down assays. **g** Western blot assays showing the IGF2BP1 protein pulled down by biotin-labeled reverse or circular RNA probes from lysates of T24T cells. **h** Cell lysates prepared from T24T cells transfected with circPTPRA or the vector were subject to immunoprecipitation with IGF2BP1 and were also hybridized with reverse or circular RNA probes for IP assays with anti-IGF2BP1 antibody, followed by western blot. **i** Cell lysates prepared from T24T cells transfected with si-circPTPRA or the mock were subject to immunoprecipitation with IGF2BP1 and were also hybridized with reverse or circular RNA probes for IP assays with anti-IGF2BP1 antibody, followed by western blot. **j** Dual RNA-FISH and immunofluorescence staining assay indicating the colocalization of circPTPRA (red) and IGF2BP1 (green) in T24T cells (scale bar represents 10 μm). **, *P* < 0.01

To explore the molecular mechanism underlying regulation of the oncogenic function of IGF2BP1 in BC, RNA immunoprecipitates (RIP) assays were performed to identify the circRNAs associated with IGF2BP1 in BC cells. As previously reported [18], sequencing purified RNA from RIP samples indicated that IGF2BP1-binding sites were enriched in protein-coding transcripts region (CDS) and the typical m^6^A modified sequences were identified (Fig. 2a, b), which was consistently supported by the previous report [18]. Meanwhile, the profile of the RIP-seq experiments identified 16 candidates for IGF2BP1-interacting circRNAs. Analysis of circRNA-seq data from three paired BC tissues and matched nontumorous tissues [23] further indicated that circPTPRA (hsa_circ_0006117) and circSMARCA5 (hsa_circ_0001445) (Fig. 2c) were the differentially-expressed circRNAs that possess potential ability to bind with IGF2BP1 in BC cells.

Moreover, knockout or overexpression of IGF2BP1 respectively attenuated or increased the interaction between IGF2BP1 and circPTPRA, but not circSMARCA5, in EJ and T24T cells (Fig. 2d&Fig. S2A), which showed the specific endogenous interaction between IGF2BP1 and circPTPRA. The small interference RNAs (siRNAs) targeting the back-splicing region of circPTPRA were established and transfected into T24T cells. It was shown that knockdown of circPTPRA significantly decreased the expression of circPTPRA, but the linear of PTPRA was not changed (Fig. S2B). Meanwhile, circPTPRA overexpression plasmid was stably transfected into T24T cells with G418 antibiotic selection (Fig. 2e). Next, RNA pull-down assay was performed with biotin-labeled sense and antisense circPTPRA RNA probes in vitro. The circular probe significantly pulled down more circPTPRA than the reverse probe (Fig. 2f). In addition, IGF2BP1 was specially pulled down by the circular probe, but not the reverse probe (Fig. 2g). Notably, the amount of IGF2BP1 pulled down by circPTPRA was significantly increased by overexpression of circPTPRA, and was decreased upon konckdown of circPTPRA (Fig. 2h, i), while the expression level of IGF2BP1 was not affected (Fig. 2h, i& Fig. S2C). Besides, the expression of circPTPRA was not affected upon overexpression or knockout of IGF2BP1 (Fig. S2D). Consistently, dual RNA-FISH and immunofluorescence assay revealed abundant signals and enrichment of circPTPRA-IGF2BP1 complex in the cytoplasm of BC cells (Fig. 2j). Taken together, these results indicated that circPTPRA was predominantly localized in the cytoplasm and could interact with IGF2BP1.

### Overexpression of circPTPRA impaired the oncogenic role of IGF2BP1 in BC both in vitro and in vivo

**Fig. 3:**
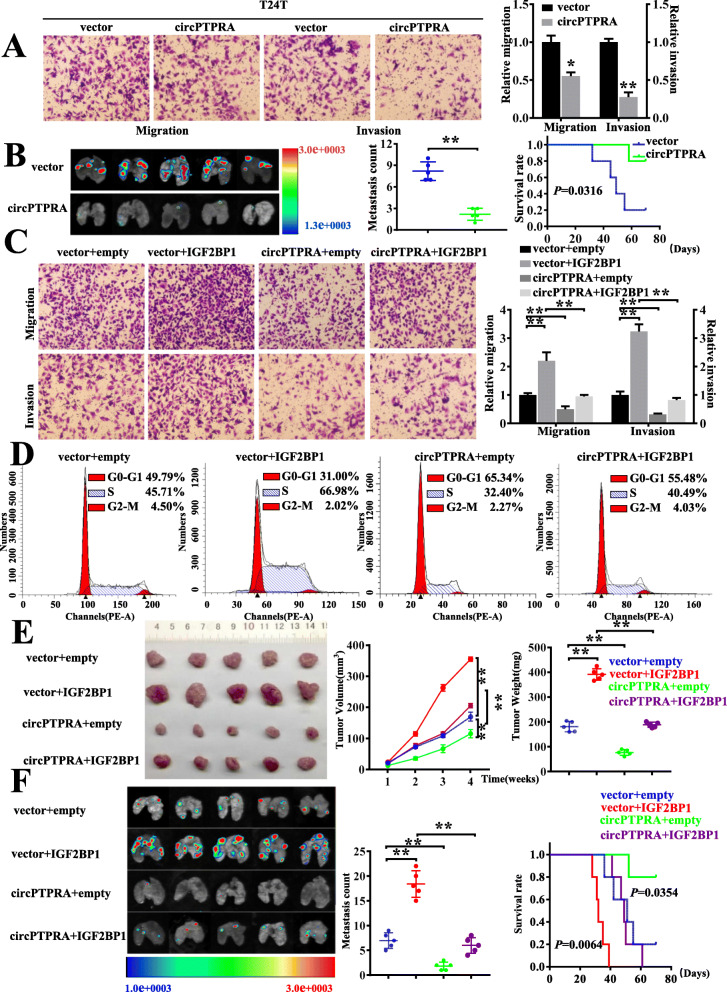
Overexpression of circPTPRA impaired the oncogenic role of IGF2BP1 in BC both in vitro and in vivo*.* **a** Representative image (left) and quantification (right) of migration and matrigel invasion assays showing the invasion of T24T cells stably transfected with vector or circPTPRA. **b** Representative images, and quantification (left) of lung metastatic colonization and Kaplan–Meier curves (right) of nude mice treated with tail-vein injection of T24T cells stably transfected with empty vector or circPTPRA (*n* = 5 for each group). **c** Representative images (left) and quantification (right) of migration and matrigel invasion assays showing the invasion of T24T cells upon ectopic expression of IGF2BP1 combined with circPTPRA overexpression. **d** Cell cycle distributions in T24T cells stably transfected as indicated were presented by flow cytometry (The results are mean ± SEM of three experiments). **e** Representative images, in vivo growth curve, and weight at the end points of subcutaneous xenograft tumors formed by T24T cells stably transfected as indicated in nude mice (*n* = 5 for each group). Student’s *t*-test, one-way ANOVA. **f** Representative fluorescence images, quantification of lung metastatic colonization, and Kaplan–Meier curves of nude mice treated with tail vein injection of T24T cells stably transfected as indicated (*n* = 5 for each group). Student’s *t*-test. Log-rank test for survival comparison. *, *P* < 0.05; **, *P* < 0.01

Previously, according to the RNA-seq, we have found that circPTPRA was down-regulated in BC tissues in comparison to paired normal tissues. It has been reported that circPTPRA inhibited BC cells proliferation by sponging miR-636 and upregulated KLF9 [24]. We confirmed that circPRPTA expression was remarkably down-regulated in BC tissues and cell lines as determined by qRT-PCR (Fig. S3A, B). Although the expression of circPTPRA had no relationship with IGF2BP1 protein (Fig. S3C), Kaplan–Meier survival curves demonstrated that low level of circPTPRA was significantly associated with poor overall survival of 64 patients with BC (Fig. S3D). Notably, in our study, we demonstrated that ectopic expression of circPTPRA significantly decreased migration and invasion capabilities of BC cell lines in vitro (Fig. 3a& Fig. S3E). In experimental metastasis assay, athymic nude mice treated with tail-vein injection of T24T cells stably transfected with circPTPRA displayed less lung metastatic colonies and greater survival probability (Fig. 3b). These results indicated that circPTPRA suppressed the invasion and metastasis of BC cells both in vivo and in vitro.

Functional assays showed that overexpression of circPTPRA significantly rescued the IGF2BP1-mediated promotion of BC cells proliferation and invasion (Fig. 3c, d& Fig. S3F, G). Consistently, in vivo assay demonstrated that overexpression of circPTPRA significantly impaired IGF2BP1-mediated promotion of subcutaneous xenograft tumor growth (Fig. 3e). Moreover, immunohistochemical staining revealed that the proliferation index Ki-67 and CD31-positive intratumoral microvessels were greatly increased in IGF2BP1-overexpressed xenograft tumors, which were attenuated by ectopic expression of circPTPRA (Fig. S3H). Importantly, athymic nude mice treated with tail vein injection of T24T cells stably transfected with IGF2BP1 displayed a higher probability of metastasis and poorer overall survival, while these effects were partly reversed by overexpression of circPTPRA (Fig. 3f). Collectively, these data indicated that circPTPRA suppressed BC progression via interacting with IGF2BP1 in vitro and in vivo.

### CircPTPRA downregulated MYC and FSCN1 expression via interacting with IGF2BP1 in BC cells

**Fig. 4:**
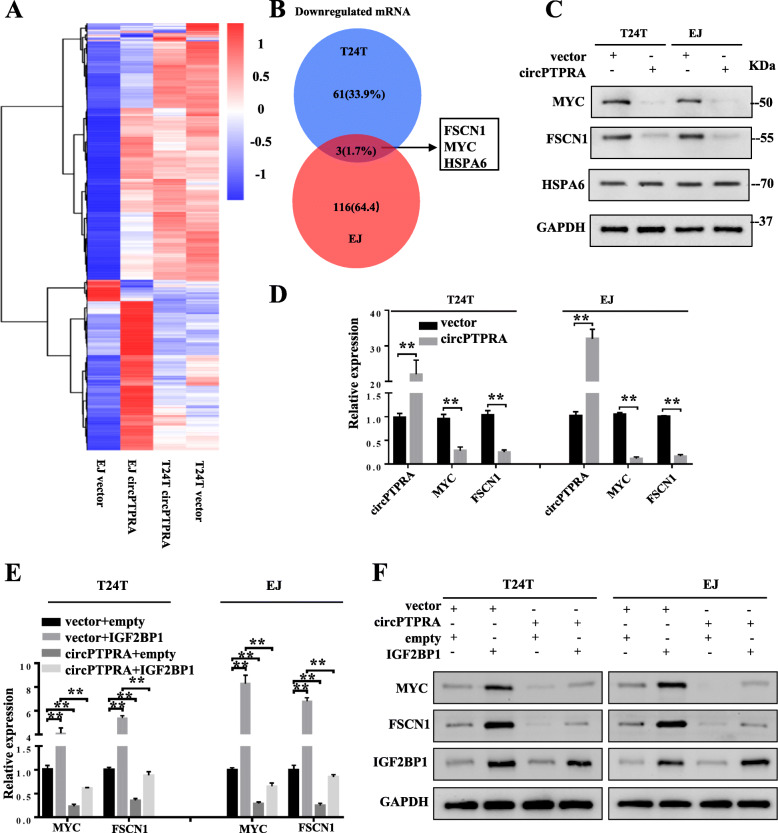
CircPTPRA downregulated MYC and FSCN1 expression via interacting with IGF2BP1 in BC cells. **a** Heat map showing the differentially expressed genes upon circPTPRA overexpression in T24T and EJ cells. Each sample contained a mixture of three repeats. **b** Venn diagram indicating the discovery of downregulated mRNAs collection in T24T and EJ cells (filtered by fold change <− 2 and *p*-value < 0.05). **c** and **d** qRT-PCR analysis (**c**) and western blot analysis (**d**) comparing circPTPRA-overexpressed BC cells with their respective control cells were shown for relative expression of MYC, FSCN1, and HSPA6. GAPDH was used as a loading control. **e** and **f** qRT-PCR analysis and western blot assay showing the expression of MYC, FSCN1, and IGF2BP1 in T24T and EJ cells stably transfected with vector or circPTPRA, and co-transfected with IGF2BP1 or control. Data are presented as the means ± SEM of three independent experiments. **, *P* < 0.01

To further investigate the target genes of circPTPRA, RNA sequencing (RNA-seq) was performed in EJ and T24T cells upon circPTPRA over-expression (Fig. 4a). It revealed that 64 mRNAs were down-regulated in T24T cells and 119 mRNAs (fold change > 2, *P* < 0.05) were reduced in EJ cells (Fig. 4b&Fig. S4A, B). Among these differentially expressed mRNAs, three mRNAs were down-regulated in both T24T and EJ cells, including MYC, FSCN1, and HSPA6 (Fig. 4b&Table. S3). Meanwhile, no protein-coding genes were found up-regulated in both cell lines (Table. S3). qRT-PCR and western blot further confirmed that the expression levels of FSCN1 and MYC mRNA were decreased in circPTPRA-overexpressed cells, whereas the expression of HSPA6 showed no significant change (Fig. 4c, d).

Notably, previous studies revealed that IGF2BP1, acted as a m^6^A reader, played an oncogenic role in cancer cells, through stabilizing methylated mRNAs of oncogenic proteins, including FSCN1, TK1, MARCKSL1, and MYC [18]. Thus, rescue experiments were performed to further investigate the interplay between circPTPRA and IGF2BP1 in regulating the expression of MYC and FSCN1. The results showed that circPTPRA significantly rescued the IGF2BP1-mediated increased expression of MYC and FSCN1 in T24T and EJ cells (Fig. 4e, f). Altogether, these results demonstrated that circPTPRA downregulated MYC and FSCN1 expression via interacting with IGF2BP1 in BC cells.

### CircPTPRA inhibits cell proliferation, migration and invasion of BC through targeting IGF2BP1/ MYC and IGF2BP1/ FSCN1 axis

**Fig. 5:**
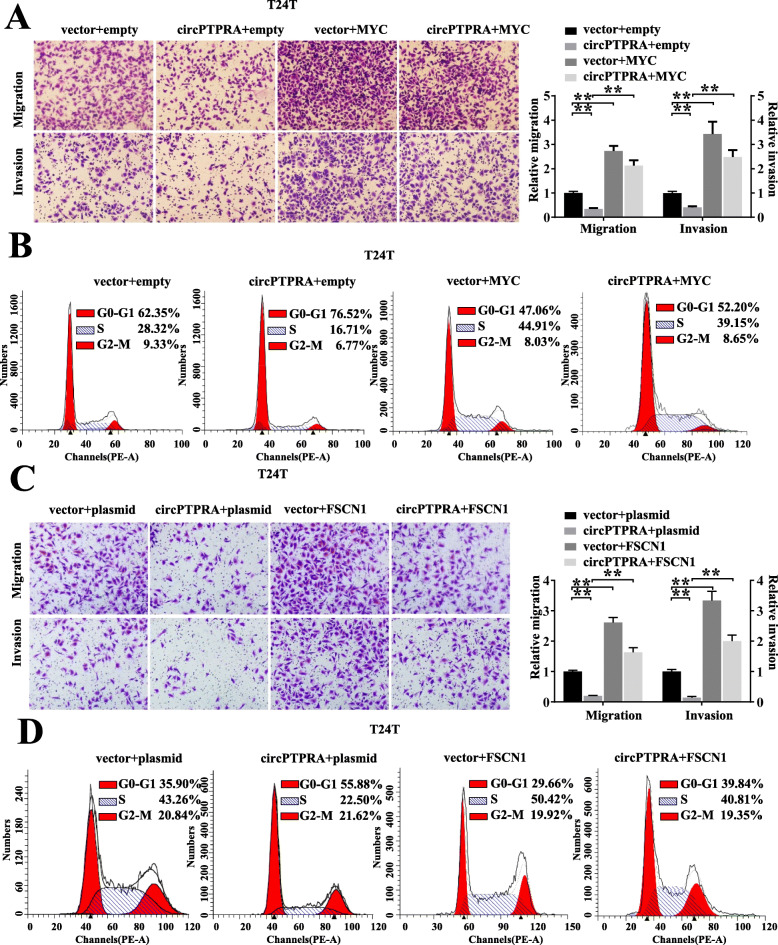
CircPTPRA inhibits cell proliferation, migration and invasion of BC through targeting IGF2BP1/ MYC and IGF2BP1/ FSCN1 axis. **a** Representative images and cell count of migration and invasion assays for circPTPRA overexpression T24T cells transiently transfected with MYC or the control. **b** Cell cycle assay of circPTPRA overexpression T24T cells transiently transfected with MYC or the control. **c** Representative images and cell counts of migration and invasion assays for circPTPRA overexpression T24T cells transiently transfected with FSCN1 or the control. **d** Cell cycle assay of circPTPRA overexpression T24T cells transiently transfected with FSCN1 or the control. Data are presented as the means ± SEM of three independent experiments. **, *P* < 0.01

Next, we investigated the functional interplay between circPTPRA and IGF2BP1/MYC, FSCN1 axis on biological features of BC cells. In vitro migration and invasion assays indicated that overexpression of MYC significantly impaired the circPTPRA-induced decreased migration and invasion abilities of BC cells (Fig. 5a&Fig. S5A). Moreover, circPTPRA-induced cell cycle arrest was partially reversed upon ectopic expression of MYC (Fig. 5b&Fig. S5B). On the other hand, enforced expression of FSCN1 also partially recue the inhibition effects of circPTPRA on cell migration and invasion, as well as cell cycle progression of BC cells (Fig. 5c, d&Fig. S5C, D). Taken together, our findings demonstrated that both MYC and FSCN1 were required for circPTPRA-mediated suppression of BC cells proliferation and invasion.

### CircPTPRA interacted with KH domains of IGF2BP1 and blocked its recognition of downstream m^6^A-modified mRNA

**Fig. 6:**
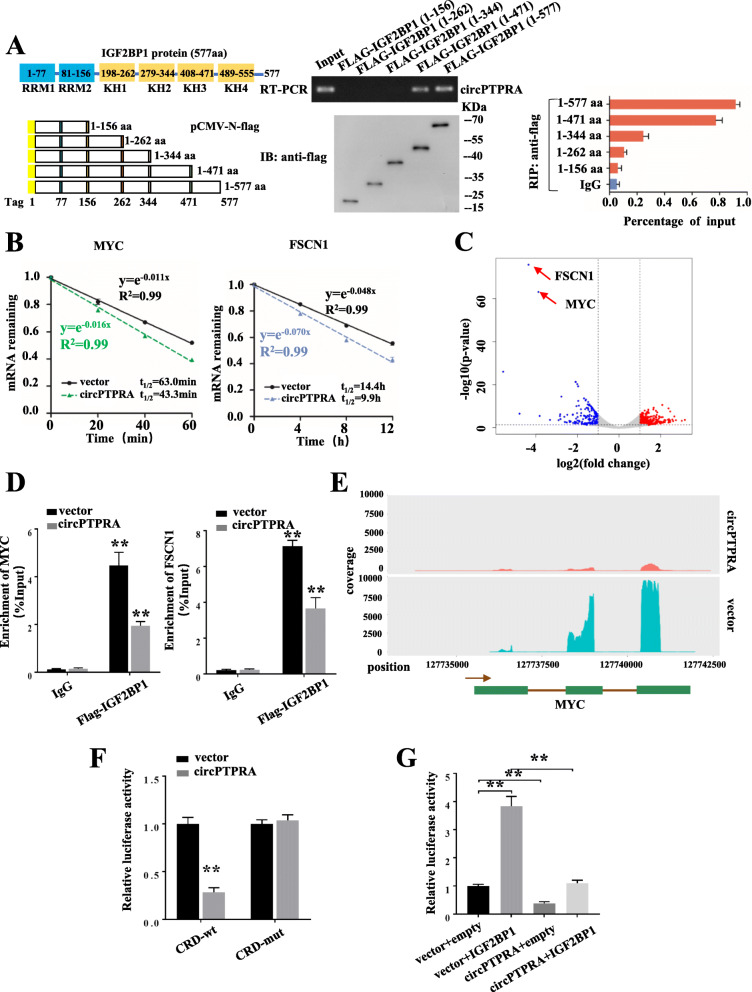
CircPTPRA affects the IGF2BP1-mediated gene regulation in an m^6^A-dependent manner. **a** In vitro binding assay showing the enriched circPTPRA levels in T24T cells detected by RT-PCR (up panel) after incubation with full-length or truncations of Flag-tagged recombinant IGF2BP1 protein validated by western blot (lower panel). RIP analysis for circPTPRA enrichment in T24T cells transiently transfected with plasmids containing the indicated FLAG-tagged full-length or truncated constructs. **b** Reducing MYC and FSCN1 mRNA half-life by overexpressing circPTPRA in T24T cells. (Values are the mean ± SD of three independent experiments) **c** RIP-seq of endogenous IGF2BP1 revealed that IGF2BP1 binding in MYC and FSCN1 transcripts in T24T cells was significantly decreased by ectopic expression of circPTPRA. **d** RIP-qPCR showed endogenous IGF2BP1 or recombinant IGF2BP1 binding in MYC and FSCN1 transcripts in T24T cells stably transfected with vector or circPTPRA. **e** RIP-seq showing the association of MYC CRD with IGF2BP1 in T24T cells stably transfected with vector or circPTPRA. **f** Relative luciferase activity of wild-type (CRD-WT) or mutated (CRD-mut) CRD reporters in 293 T cells with or without ectopic expression of circPTPRA. **g** Relative luciferase activity of CRD-WT in IGF2BP1 overexpression or control 293 T cells with or without ectopic expression of circPTPRA. Values are the mean ± SD of three independent experiments, and two-tailed Student’s *t*-tests were used in **f**-**g**. **, *P* < 0.01

Subsequently, we further elucidate the mechanisms underlying circPTPRA regulation of IGF2BP1 function. In vitro binding assay showed that deletion of RNA-binding K homology (KH) domains KH3 and KH4 (405–553 amino acids) of FLAG-tagged IGF2BP1 protein, but not other domains, significantly abolished the interaction between IGF2BP1 and circPTPRA, indicating that KH3 and KH4 domains of IGF2BP1 was crucial for its interaction with circPTPRA (Fig. 6a). Since KH domains of IGF2BP1 play important roles in regulation of mRNA stability, mRNA decay assay of MYC and FSCN1 was applied upon overexpression of circPTPRA, and accelerated mRNA decay rates were found in T24T cells (Fig. 6b). Sequencing of RNA immunoprecipitates (RIPs) of endogenous IGF2BP1 revealed that IGF2BP1 binding in MYC and FSCN1 transcripts in T24T cells were significantly decreased by ectopic expression of circPTPRA (Fig. 6c&Table. S4). Similar results were observed in RIP assays with FLAG-tagged IGF2BP1 (Fig. 6d).

The coding region instability determinant (CRD)-containing region in the 3′-terminus of the MYC mRNA coding region has a high abundance of m^6^A modifications and has been proved critical for IGF2BP1 binding [18]. Further analysis of the RIP-sequencing results revealed that ectopic expression of circPTPRA significantly reduced the cellular IGF2BP1 binding in MYC CRD region (Fig. 6e). We next inserted the 249-nt wild-type or mutant CRD sequence of MYC into a firefly luciferase (Fluc) reporter. Luciferase reporter assays showed that the relative luciferase activity of reporters with wild-type CRD, but not those with mutant CRD, was decreased by circPTPRA overexpression (Fig. 6f). Furthermore, IGF2BP1-mediated increase of luciferase activity of MYC CRD region could be blocked by enforced expression of circPTPRA (Fig. 6g).

**Fig. 7:**
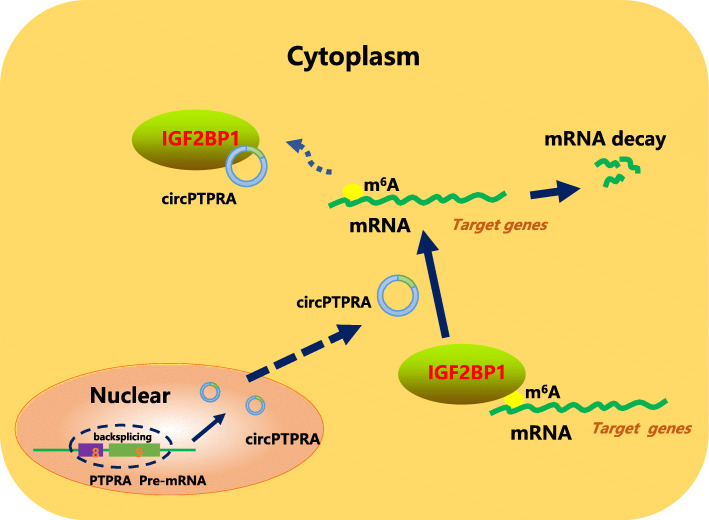
Schematic diagram of the circPTPRA-regulated pathway in bladder cancer cells

Taken together, our data indicated that circPTPRA could directly bind to the KH3 and KH4 domains of IGF2BP1 and block its recognition of downstream m^6^A-modified mRNAs, thereby decrease the stability of the target mRNAs (Fig. 7).

## Discussion

Recent studies have identified IGF2BPs as novel carcinogenesis factors in a number of solid tumors, including ovarian, breast, melanoma and hepatocellular tumors, and its high expression is associated with metastasis and poor prognosis [25]. Whereas the oncogenic role IGF2BP1 and its paralogs in BC remains unknown. In this study, our results indicated that high expression of IGF2BP1 was associated with poor prognosis in BC. As a RNA-binding protein, IGF2BP1 apparently ‘cages’ their target mRNAs in cytoplasmic protein–RNA complexes, preventing the premature decay of specific target transcripts in an RNA-dependent manner [26]. The stable ‘caging’ of transported mRNAs allows for their ‘long-distance’ transport during cellular stress as well as transient storage [27]. In this study, our RIP-Seq data revealed that IGF2BP1-binding sites were enrichment in protein-coding transcripts region (CDS) and m^6^A-dependent binding mode was on top of the primary sequence. Our gain- and loss-of-function studies indicated that IGF2BP1 facilitated the proliferation, migration and invasion of cancer cells, suggesting the oncogenic roles of IGF2BP1 in BC. These findings indicate that IGF2BP1 could be served as therapeutic target, as well as possibly be used for potential clinical diagnosis and prognosis evaluation of BC.

The most prevalent RNA methylation, *N*^6^-methyladenosine (m^6^A), occurs in approximately 25% of transcripts at the genome-wide level and is enriched in 5′- and 3′ -untranslated regions. m^6^A is installed by m^6^A methyltransferases (METTL3/14, WTAP), reverted by m^6^A demethylases (FTO, ALKBH5) and recognized by reader proteins (YTHDF1/2/3, IGF2BPs) [28–30]. Accumulating evidences show that, m^6^A RNA methylation has an outsize effect on RNA production/metabolism and participates in the pathogenesis of multiple diseases including cancers [31]. As a key m^6^A reader, the transcription of IGF2BP1 is modulated by negative as well as positive feed-back regulation of proteins, including b-catenin (CTNNB1) and MYC [32, 33]. The up-regulated three lncRNAs (HCG11, GHET1, and THOR) can elevate IGF2BP1 level, potentially via forming the surprisingly long half-life of IGF2BP-RNA complexes [21, 34, 35]. Besides, LncRNA LIN28B-AS1 could alter the LIN28B mRNA stability by physical combination with IGF2BP1 [36]. In this study, the oncogenic function of IGF2BP1 in BC growth and aggressiveness was reversed by ectopic expression of circPTPRA in vivo and in vitro through reducing its interaction with downstream target m^6^A-modified mRNA, while the expression level of IGF2BP1 was not affected. These results extend our knowledge about the regulation mode of IGF2BP1 function in cancer cells, which is mediated by the direct binding of circRNA with IGF2BP1. Enhancing the effect of tumor suppressive circRNAs, such as circPTPRA, may act as efficient therapeutic strategies for future cancer therapy. Exogenous circRNAs could be achieved by gene therapy where DNA cassettes designed for circRNAs expression are delivered, or by transfection of purified, in vitro-generated circRNAs [37]. These stably transfected circRNAs produce more quantity of proteins than modified linear mRNA or unmodified counterparts.

IGF2BP1 consists of six canonical RNA-binding domains, including two RNA-recognition motifs (RRMs) in the *N*-terminal part and four hnRNP-K homology (KH) domains in the *C*-terminal region [38]. In vitro studies revealed that the stabilization of IGF2BP1-RNA complexes is mainly facilitated via the KH3/4 domain, which could potentially contribute to the binding of IGF2BP1 to the MYC-CRD (coding region stability determinant) RNA [18, 39]. In this study, we found that KH3/4 domain of IGF2BP1 was also necessary for its interaction with circPTPRA, and circPTPRA promoted the endonuclease-directed decay of downstream mRNAs via forming a circRNA/IGF2BP1 complex to sequester the transcripts. This mode of regulation is presumably relied on the affinity of the association between circRNA and IGF2BP1, resulting in the competitive interaction of circRNA and target mRNAs with IGF2BP1. It has been previously reported that circNSUN2 could enhance the stability of HMGA2 mRNA to promote colorectal carcinoma metastasis progression by forming a circNSUN2/IGF2BP2/HMGA2 RNA-protein ternary complex in the cytoplasm [40]. Here, we revealed a different role of circRNA in regulation of IGF2BP1 function, which acts as an effective endogenous blocker.

Silencing of IGF2BP1 globally down-regulates target gene expression in mRNA level, including FSCN1, TK1, MARCKSL1, and MYC [18]. Recent studies have shown an association between the up-regulated expression of FSCN1 and increased invasiveness of carcinomas in the urinary bladder, which suggests that FSCN1 may be a marker of aggressive bladder cancer [41, 42]. The expression of FSCN1 could be indirectly regulated by lncRNA through “miRNA sponge” effect at the transcriptional level, including lncRNA-UCA1, LINC00152 and ZEB1-AS1 [43–45]. Oncogene MYC is known to be aberrantly expressed in BC and acts as a master regulator of genes involved in cell cycle progression, cell growth, differentiation, metabolism, and apoptosis [46]. Mechanisms of MYC deregulation in BC include signal transduction transcriptional regulation [47], miRNA mediated post-transcriptional regulation [48] and DNA mutation [49]. Besides, alteration of m^6^A levels also participates in cancer pathogenesis via regulating expression of MYC. For example, depletion of METTL3 in BC cells decreased the stability of MYC transcripts through affecting m^6^A abundance mainly around the stop codon and 3′-UTR regions [50]. *N*^6^-methyladenosine modification in the CRD of the MYC mRNA enhances the association of IGF2BPs and interferes with the endonuclease-directed decay of the MYC mRNA [51]. In the current study, we found that FSCN1 and MYC, but not other targets, were involved in circPTPRA/IGF2BP1 regulation of m^6^A recognition and mRNA stability. Our observations indicate that the downstream effectors of IGF2BPs recognition of m^6^A have cell- and tissue- specificities, and the targets of each circRNA and IGF2BPs complex need to be individually identified.

## Conclusions

In summary, our data demonstrate that circPTPRA is an important tumor suppressor for BC. Functionally, circPTPRA suppressed the proliferation, migration and invasion of BC cells in vivo and in vitro. Mechanistically, circPTPRA could interact with IGF2BP1 and block the recognition of IGF2BP1 to m^6^A-modified RNAs, resulting in downregulation of FSCN1 and MYC mRNA stability. Our study clarified that circPTPRA acted as a potential endogenous blocker and broadened the options for curative management of BC.

## Supplementary Information

**Additional file 1: Figure S1.** A. and B. The efficiencies of stable models of IGF2BP1 overexpression or knockout in EJ and T24T cell lines. C. Sanger sequencing of genomic DNAs to validate IGF2BP1 knockout in EJ and T24T cells. The mutation patterns on the two alleles are highlighted in red. The red arrow represents the cleavage site. D. CCK-8 assay was performed to evaluate cell viability in BC cells with stable overexpression or knockout of IGF2BP1. E. and F. Cell cycle analysis and representative images and quantification of transwell assay indicating the proliferation and invasion of EJ cells with stable overexpression or knockout of IGF2BP1. The data are the means ± SEM of three independent experiments. **, *P* < 0.01. **Figure S2.** A. RIP and qRT-PCR assays using an antibody specific for IGF2BP1 showing the interaction between circRNAs (circPTPRA, circSMARCA5) and IGF2BP1 in EJ cells with stable overexpression or knockout of IGF2BP1. B. The knockdown efficiency of circPTPRA in T24T cells transfected with circPTPRA siRNA or corresponding negative control were evaluated by qRT-PCR. C. Overexpression or knockdown of circPTPRA did not affect the IGF2BP1 expression levels in T24T cells. D. Overexpression or knockout of IGF2BP1 did not affect the circPTPRA expression levels in T24T cells. **, *P* < 0.01. **Figure S3.** A. The expression level of circPTPRA in 64 paired of BC and corresponding adjacent tissues was determined by qRT-PCR. GAPDH was used as a loading control. B. The relative expression of circPTPRA was detected using qRT-PCR in SV-HUC-1, RT4, UMUC3, EJ, T24T and 5637 cells. GAPDH was used as endogenous control. C. The correlation between the transcript levels of IGF2BP1 and circPTPRA by qRT-PCR in BC tissues (*n* = 64). D. Kaplan-Meier curves indicating overall survival of 64 bladder cancer patients with ow or high expression of circPTPRA (the patients were divided into high- and low-expression groups using the median value as the cut-off point, *n* = 64, *p* = 0.0009, log-rank test). E. Representative image (left) and quantification (right) of migration and matrigel invasion assays showing the invasion of EJ cells stably transfected with empty vector and circPTPRA. F. Representative images (left) and quantification (right) of migration and matrigel invasion assays showing the invasion of EJ cells upon ectopic expression of IGF2BP1 combined with circPTPRA overexpression. **G.** Cell cycle distributions in EJ cells stably transfected as indicated were presented by flow cytometry (The results are mean ± SEM of three experiments). H. Immunohistochemical staining and quantification showing the Ki-67 and CD31 expression within subcutaneous xenograft tumors formed by T24T cells stably transfected as indicated (*n* = 5 for each group). Scale bars: 100 μm. Pearson’s correlation coefficient analysis in Figure S3C. **, *P* < 0.01. **Figure S4.** A. and B. The volcano plots in EJ and T24T cells upon circPTPRA overexpression. **Figure S5.** A. Representative images and cell count of migration and invasion assays for circPTPRA overexpression EJ cells transiently transfected with MYC or the control. B. Cell cycle assay of circPTPRA overexpression EJ cells transiently transfected with MYC or the control. C. Representative images and cell counts of migration and invasion assays for circPTPRA overexpression EJ cells transiently transfected with FSCN1 or the control. D. Cell cycle assay of circPTPRA overexpression EJ cells transiently transfected with FSCN1 or the control. Data are presented as the means ± SEM of three independent experiments. **, *P* < 0.01.

**Additional file 2: Table S1.** Clinicopathological features of 64 BC patients and the expression of IGF2BP1 and circPTPRA. **Table S2.** Detailed information of primers and RNA sequences used in this study. **Table S3.** The differentially expressed protein-coding genes in EJ and T24T cell lines upon circPTPRA overexpression. **Table S4.** The differentially immunoprecipitated transcripts that interacted with IGF2BP1 in T24T cells upon ectopic expression of circPTPRA.

## Data Availability

The datasets supporting the conclusions of this article are included within the article and its additional files.

## Abbreviations

CircRNAs: Circular RNAs
BC: Bladder cancer
IGF2BP1: Insulin-like growth factor-2 mRNA-binding protein 1
RIP-seq: RNA immunoprecipitation sequencing
m^6^A: *N*^*6*^-methyladenosine
miRNA: microRNA
FAAH: Fatty acid amide hydrolase
RIP: RNA Immunoprecipitation
TCGA: The Cancer Genome Atlas
CDS: Protein-coding transcripts region
