# Correction: CircPTPRA blocks the recognition of RNA *N*^*6*^-methyladenosine through interacting with IGF2BP1 to suppress bladder cancer progression

**DOI:** 10.1186/s12943-022-01677-8

**Published:** 2022-11-01

**Authors:** Fei Xie, Chao Huang, Feng Liu, Hui Zhang, Xingyuan Xiao, Jiayin Sun, Xiaoping Zhang, Guosong Jiang

**Affiliations:** 1 Department of Urology, Union Hospital, Tongji Medical College, Huazhong University of Science and Technology, Wuhan, 430022 China; 2 Department of Urology, The Affiliated Hospital of Qingdao University, Qingdao, 266013 China

**Correction: Mol Cancer 20, 68 (2021)**

**https://doi.org/10.1186/s12943-021-01359-x**

Following the publication of the original paper [1], we have found several inadvertent errors in the figures recently. Those errors are as following:

The images for transwell assays in Fig. 1G (invasion assay for “IGF2BP1 group” and “sgCtrl group”), Fig. 3A (invasion assay for “vector group”), Fig. 3C (migration assay for “vector/IGF2BP1 group”, “circPTPRA/empty group”, and “circPTPRA/IGF2BP1 group”; invasion assay for “vector/empty group”, “vector/IGF2BP1 group”, “circPTPRA/empty group”, and “circPTPRA/IGF2BP1 group”), Fig. 5A (migration assay for “vector/empty group”, “circPTPRA/empty group”, “vector/MYC group”, and “circPTPRA/MYC group”), and Fig. S1F (migration assay for “IGF2BP1 group”; invasion assay for “empty group”, “sgCtrl group”, and “sgIGF2BP1 group”) were misused. After checking the original data, we found that these errors happened inadvertently during the stage of saving the images, partially due to two projects were conducted simultaneously. Besides, the image for lung metastatic colonization in Fig. 1I (“sgIGF2BP1#1 group” No.1) was misused, and the MYC and FSCN1 mRNA half-life in Fig. 6B were mistakenly displayed. The XY axis labels in Fig. S1E were misplaced. The corrected version of Fig. 1G&I, Fig. 3A&C, Fig. 5A, Fig. 6B and Fig. S1E&F have been provided. Besides, we made a mistake to describe “male nude mice” as “female nude mice” in the Methods. Since these errors do not originate from original experiments, there is no effect on the interpretation or conclusion of this work. We sincerely apologize to the editor, reviewers and readers for the errors and any confusion it may have caused.

**Fig. 1:**
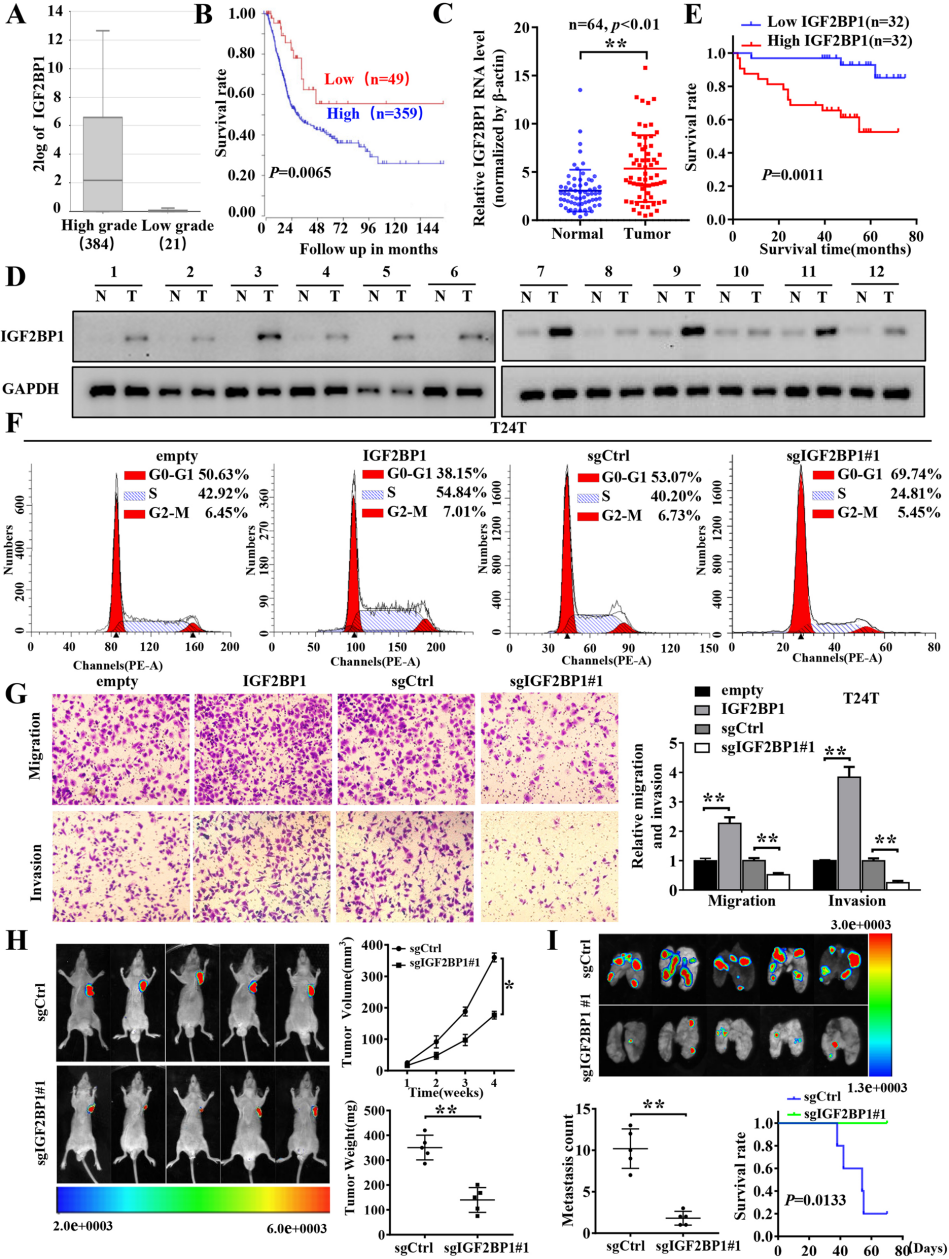
IGF2BP1 was up-regulated in BC and could promote BC cells progression in vitro and in vivo. **a** The mRNA expression levels of IGF2BP1 in BC obtained from TCGA datasets. **b** The Kaplan–Meier curves with univariate analyses of overall survival (OS) in BC patients with low versus high expression of IGF2BP1 from TCGA cohorts. **c** The expression of IGF2BP1 in BC tissues (Tumor) compared to paired adjacent normal bladder tissues (Normal) of 64 clinical patients. **d** Western blot indicating IGF2BP1 protein levels were significantly upregulated in BC tissues compared with adjacent normal bladder epithelial tissues. **e** Kaplan–Meier’s analyze of correlation between IGF2BP1 expression level and overall survival of 64 patients with BC (the patients were divided into high- and low-expression groups using the median value as the cut-off point, *n* = 64, *p* = 0.0011, log-rank test). **f** and **g** Cell cycle analysis (**f**) and representative images (left) and quantification (right) of transwell assay (**g**) indicating the proliferation and invasion of T24T cells with stable overexpression or knockout of IGF2BP1. **h** Representative images, in vivo growth curve (bottom right, up), and weight at the end points (bottom right, down) of xenografts formed by subcutaneous injection of T24T cells transfected with CRISPR-vector, or CRISPR Cas9-IGF2BP1 into the dorsa flanks of nude mice (*n* = 5 for each group). **i** Representative images, and quantification (bottom left) of lung metastatic colonization and Kaplan–Meier curves (bottom right) of nude mice treated with tail-vein injection of T24T cells stably transfected with sgCtrl or sgIGF2BP1#1 (*n* = 5 for each group). *, *P* < 0.05; **, *P* < 0.01

**Fig. 3:**
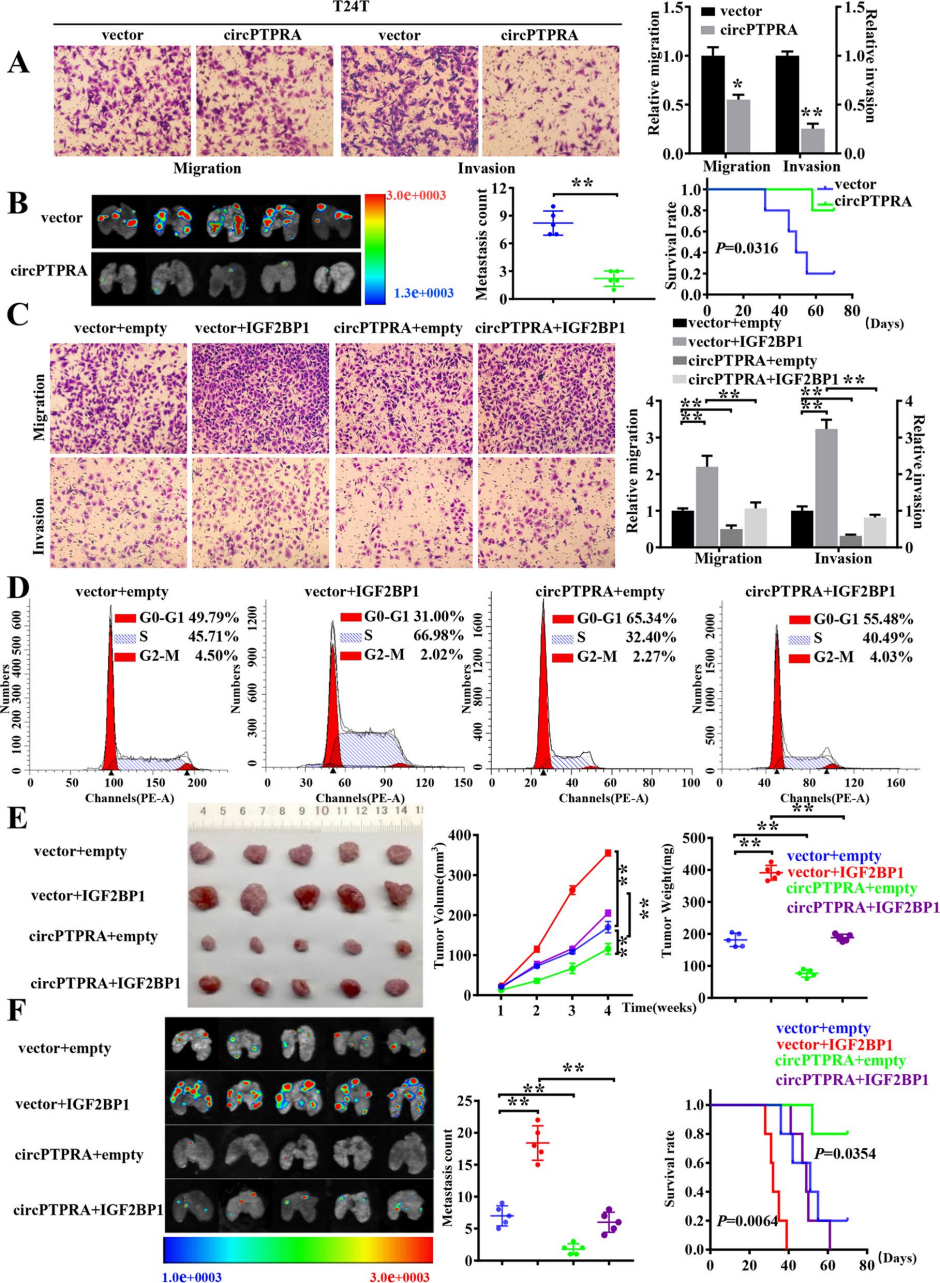
Overexpression of circPTPRA impaired the oncogenic role of IGF2BP1 in BC both in vitro and in vivo*.* **a** Representative image (left) and quantification (right) of migration and matrigel invasion assays showing the invasion of T24T cells stably transfected with vector or circPTPRA. **b** Representative images, and quantification (left) of lung metastatic colonization and Kaplan–Meier curves (right) of nude mice treated with tail-vein injection of T24T cells stably transfected with empty vector or circPTPRA (*n* = 5 for each group). **c** Representative images (left) and quantification (right) of migration and matrigel invasion assays showing the invasion of T24T cells upon ectopic expression of IGF2BP1 combined with circPTPRA overexpression. **d** Cell cycle distributions in T24T cells stably transfected as indicated were presented by flow cytometry (The results are mean ± SEM of three experiments). **e** Representative images, in vivo growth curve, and weight at the end points of subcutaneous xenograft tumors formed by T24T cells stably transfected as indicated in nude mice (*n* = 5 for each group). Student’s *t*-test, one-way ANOVA. **f** Representative fluorescence images, quantification of lung metastatic colonization, and Kaplan–Meier curves of nude mice treated with tail vein injection of T24T cells stably transfected as indicated (*n* = 5 for each group). Student’s *t*-test. Log-rank test for survival comparison. *, *P* < 0.05; **, *P* < 0.01

**Fig. 5:**
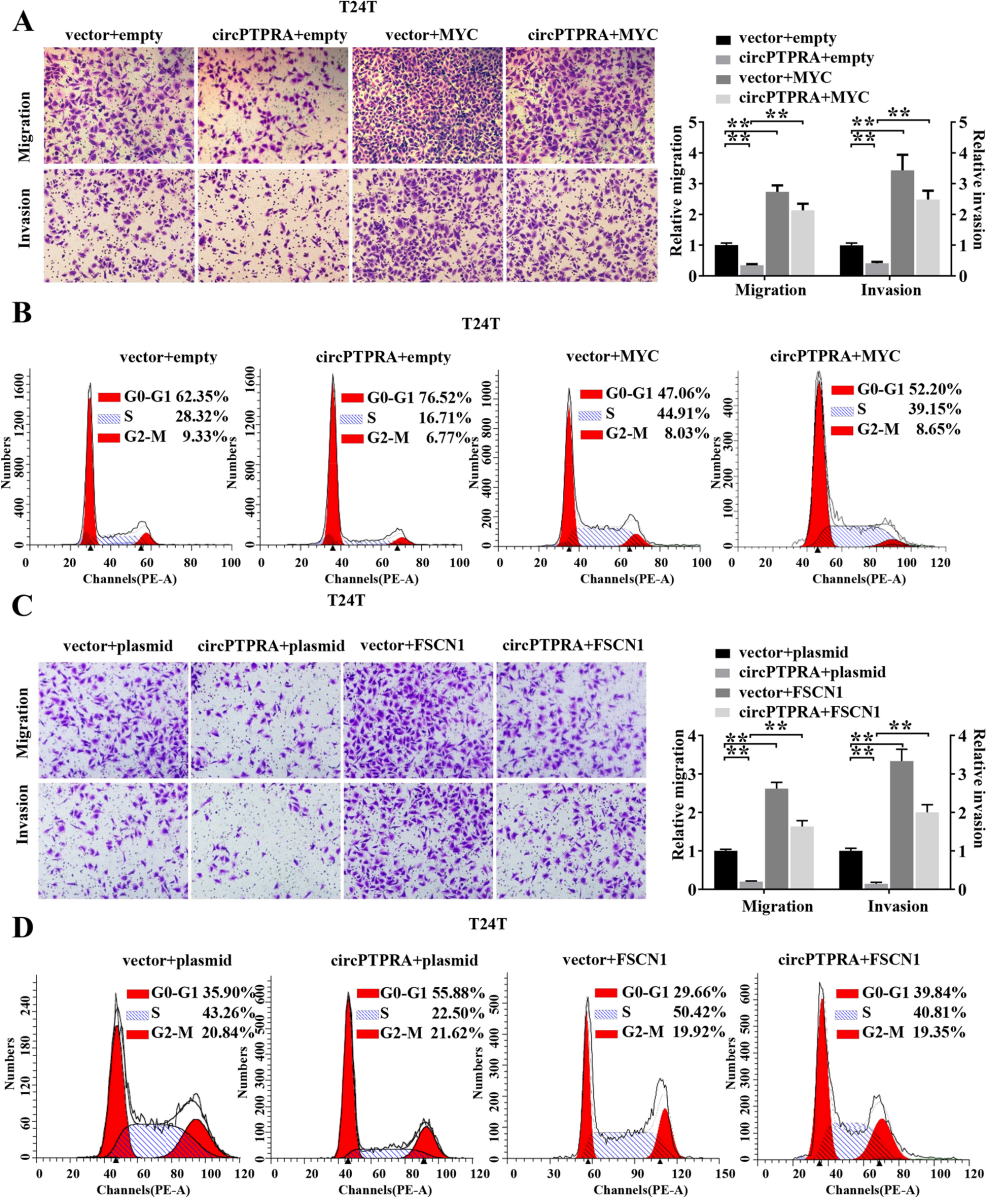
CircPTPRA inhibits cell proliferation, migration and invasion of BC through targeting IGF2BP1/ MYC and IGF2BP1/ FSCN1 axis. **a** Representative images and cell count of migration and invasion assays for circPTPRA overexpression T24T cells transiently transfected with MYC or the control. **b** Cell cycle assay of circPTPRA overexpression T24T cells transiently transfected with MYC or the control. **c** Representative images and cell counts of migration and invasion assays for circPTPRA overexpression T24T cells transiently transfected with FSCN1 or the control. **d** Cell cycle assay of circPTPRA overexpression T24T cells transiently transfected with FSCN1 or the control. Data are presented as the means ± SEM of three independent experiments. **, *P* < 0.01

**Fig. 6:**
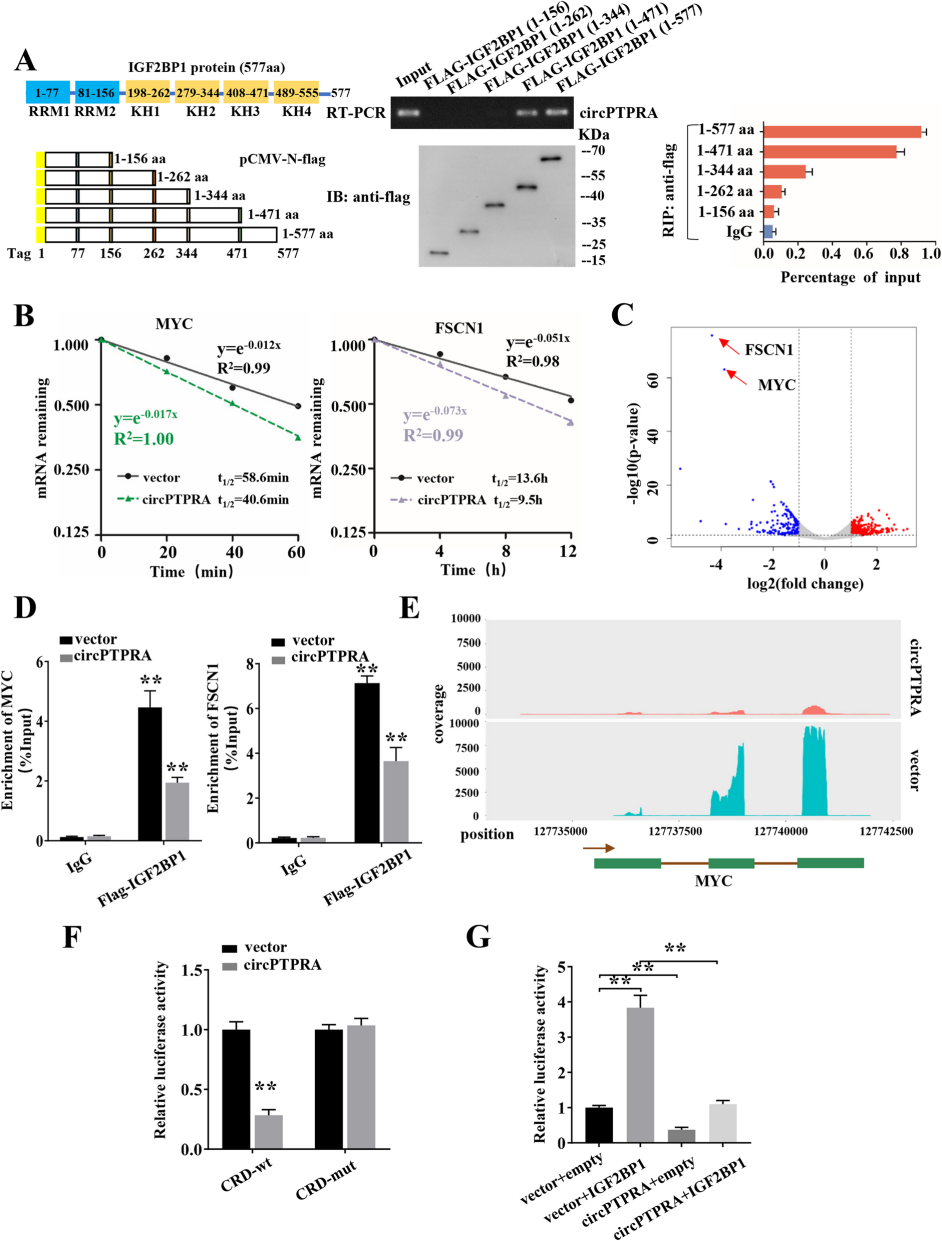
CircPTPRA affects the IGF2BP1-mediated gene regulation in an m^6^A-dependent manner. **a** In vitro binding assay showing the enriched circPTPRA levels in T24T cells detected by RT-PCR (up panel) after incubation with full-length or truncations of Flag-tagged recombinant IGF2BP1 protein validated by western blot (lower panel). RIP analysis for circPTPRA enrichment in T24T cells transiently transfected with plasmids containing the indicated FLAG-tagged full-length or truncated constructs. **b** Reducing MYC and FSCN1 mRNA half-life by overexpressing circPTPRA in T24T cells. (Values are the mean ± SD of three independent experiments) **c** RIP-seq of endogenous IGF2BP1 revealed that IGF2BP1 binding in MYC and FSCN1 transcripts in T24T cells was significantly decreased by ectopic expression of circPTPRA. **d** RIP-qPCR showed endogenous IGF2BP1 or recombinant IGF2BP1 binding in MYC and FSCN1 transcripts in T24T cells stably transfected with vector or circPTPRA. **e** RIP-seq showing the association of MYC CRD with IGF2BP1 in T24T cells stably transfected with vector or circPTPRA. **f** Relative luciferase activity of wild-type (CRD-WT) or mutated (CRD-mut) CRD reporters in 293 T cells with or without ectopic expression of circPTPRA. **g** Relative luciferase activity of CRD-WT in IGF2BP1 overexpression or control 293 T cells with or without ectopic expression of circPTPRA. Values are the mean ± SD of three independent experiments, and two-tailed Student’s *t*-tests were used in **f**-**g**. **, *P* < 0.01

## Supplementary Information

**Additional file 1: Figure S1.** A. and B. The efficiencies of stable models of IGF2BP1 overexpression or knockout in EJ and T24T cell lines. C. Sanger sequencing of genomic DNAs to validate IGF2BP1 knockout in EJ and T24T cells. The mutation patterns on the two alleles are highlighted in red. The red arrow represents the cleavage site. D. CCK-8 assay was performed to evaluate cell viability in BC cells with stable overexpression or knockout of IGF2BP1. E. and F. Cell cycle analysis and representative images and quantification of transwell assay indicating the proliferation and invasion of EJ cells with stable overexpression or knockout of IGF2BP1. The data are the means ± SEM of three independent experiments. **, *P* < 0.01.
