# Supplementary material for: CircPTPRA blocks the recognition of RNA N6-methyladenosine through interacting with IGF2BP1 to suppress bladder cancer progression
Source: Mol Cancer. 2021 Apr 14;20:68. doi: 10.1186/s12943-021-01359-x (PMC8045402; doi:10.1186/s12943-021-01359-x)
Supplement: Supplementary file 1 — Additional file 1: Figure S1. A. and B. The efficiencies of stable models of IGF2BP1 overexpression or knockout in EJ and T24T cell lines. C. Sanger sequencing of genomic DNAs to validate IGF2BP1 knockout in EJ and T24T cells. The mutation patterns on the two alleles are highlighted in red. The red arrow represents the cleavage site. D. CCK-8 assay was performed to evaluate cell viability in BC cells with stable overexpression or knockout of IGF2BP1. E. and F. Cell cycle analysis and representative images and quantification of transwell assay indicating the proliferation and invasion of EJ cells with stable overexpression or knockout of IGF2BP1. The data are the means ± SEM of three independent experiments. **, P < 0.01. Figure S2. A. RIP and qRT-PCR assays using an antibody specific for IGF2BP1 showing the interaction between circRNAs (circPTPRA, circSMARCA5) and IGF2BP1 in EJ cells with stable overexpression or knockout of IGF2BP1. B. The knockdown efficiency of circPTPRA in T24T cells transfected with circPTPRA siRNA or corresponding negative control were evaluated by qRT-PCR. C. Overexpression or knockdown of circPTPRA did not affect the IGF2BP1 expression levels in T24T cells. D. Overexpression or knockout of IGF2BP1 did not affect the circPTPRA expression levels in T24T cells. **, P < 0.01. Figure S3. A. The expression level of circPTPRA in 64 paired of BC and corresponding adjacent tissues was determined by qRT-PCR. GAPDH was used as a loading control. B. The relative expression of circPTPRA was detected using qRT-PCR in SV-HUC-1, RT4, UMUC3, EJ, T24T and 5637 cells. GAPDH was used as endogenous control. C. The correlation between the transcript levels of IGF2BP1 and circPTPRA by qRT-PCR in BC tissues (n = 64). D. Kaplan-Meier curves indicating overall survival of 64 bladder cancer patients with ow or high expression of circPTPRA (the patients were divided into high- and low-expression groups using the median value as the cut-off point, n = 64, p = 0.0009, lo [file 12943_2021_1359_MOESM1_ESM.zip › Fig S1.pdf]

Fig S1

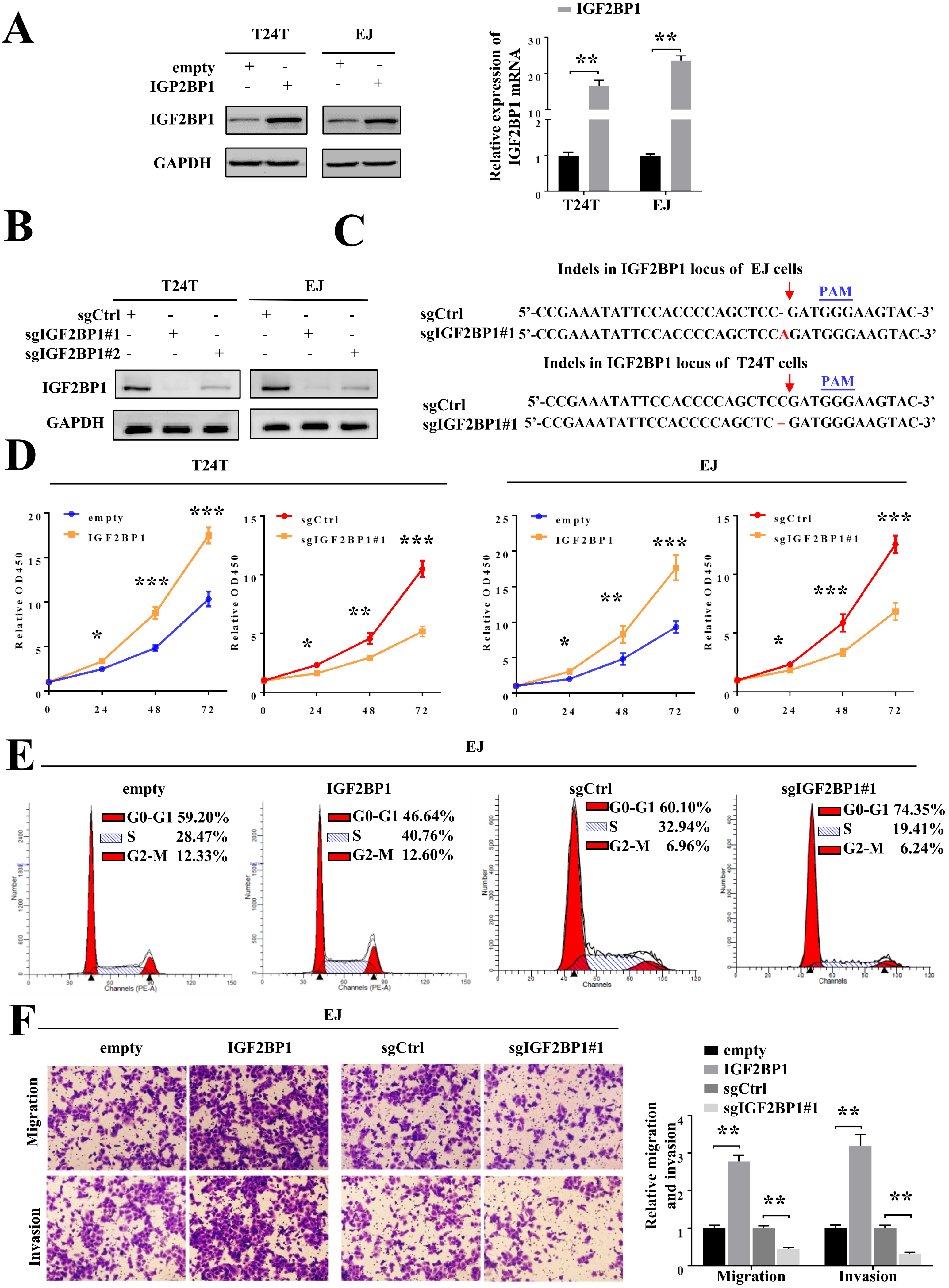

A
T24T
EJ
empty
IGP2BP1
IGF2BP1
GAPDH
empty
IGF2BP1
Relative expression of IGF2BP1 mRNA
T24T
EJ
**
B
T24T
EJ
sgCtrl
sgIGF2BP1#1
sgIGF2BP1#2
IGF2BP1
GAPDH
C
Indels in IGF2BP1 locus of EJ cells
PAM
sgCtrl 5'-CCGAAATATTCCACCCCAGCTCC- GATGGGAAGTAC-3'
sgIGF2BP1#1 5'-CCGAAATATTCCACCCCAGCTCCAGATGGGAAGTAC-3'
Indels in IGF2BP1 locus of T24T cells
PAM
sgCtrl 5'-CCGAAATATTCCACCCCAGCTCCGATGGGAAGTAC-3'
sgIGF2BP1#1 5'-CCGAAATATTCCACCCCAGCTC – GATGGGAAGTAC-3'
D
T24T
EJ
empty
IGF2BP1
sgCtrl
sgIGF2BP1#1
Relative OD450
E
EJ
empty
G0-G1 59.20%
S 28.47%
G2-M 12.33%
IGF2BP1
G0-G1 46.64%
S 40.76%
G2-M 12.60%
sgCtrl
G0-G1 60.10%
S 32.94%
G2-M 6.96%
sgIGF2BP1#1
G0-G1 74.35%
S 19.41%
G2-M 6.24%
Channels (PE-A)
Number
F
EJ
empty
IGF2BP1
sgCtrl
sgIGF2BP1#1
Migration
Invasion
Relative migration and invasion
