# Supplementary material for: CircPTPRA blocks the recognition of RNA N6-methyladenosine through interacting with IGF2BP1 to suppress bladder cancer progression
Source: Mol Cancer. 2021 Apr 14;20:68. doi: 10.1186/s12943-021-01359-x (PMC8045402; doi:10.1186/s12943-021-01359-x)
Supplement: Supplementary file 2 — Additional file 2: Table S1. Clinicopathological features of 64 BC patients and the expression of IGF2BP1 and circPTPRA. Table S2. Detailed information of primers and RNA sequences used in this study. Table S3. The differentially expressed protein-coding genes in EJ and T24T cell lines upon circPTPRA overexpression. Table S4. The differentially immunoprecipitated transcripts that interacted with IGF2BP1 in T24T cells upon ectopic expression of circPTPRA. [file 12943_2021_1359_MOESM2_ESM.zip › Table. S1.pdf]

Parameters	Group	Cases	IGF2BP1 expression				P value	circPTPRA expression				P value
			Low	%	High	%		Low	%	High	%	
Gender	Male	45	9	20	36	80	0.5766	35	78	10	22	0.2263
	Female	19	5	26	14	74		12	63	7	37	
Age(years)	<55	28	6	21	22	79	0.9393	20	71	8	29	0.7483
	≥55	36	8	22	28	78		27	75	9	25	
Tumor stage	pTa-T1	15	8	53	7	47	0.0008	7	47	8	53	0.0073
	pT2-T4	49	6	12	43	88		40	82	9	18	
Tumor size	<3.0 cm	18	5	28	13	72	0.4749	9	50	9	50	0.0079
	≥3.0cm	46	9	20	37	80		38	83	8	17	
Grade	Low	13	7	54	6	46	0.0018	6	46	7	54	0.0126
	High	51	7	14	44	86		41	80	10	20	
Lymph node metastasis	Absent	42	13	31	29	69	0.0350	26	62	16	38	0.0096
	Present	22	1	5	21	95		21	95	1	5	

$P < 0.05$ represents statistical significance (Chi-square test).
