# Supplementary material for: CircPTPRA blocks the recognition of RNA N6-methyladenosine through interacting with IGF2BP1 to suppress bladder cancer progression
Source: Mol Cancer. 2021 Apr 14;20:68. doi: 10.1186/s12943-021-01359-x (PMC8045402; doi:10.1186/s12943-021-01359-x)
Supplement: Supplementary file 2 — Additional file 2: Table S1. Clinicopathological features of 64 BC patients and the expression of IGF2BP1 and circPTPRA. Table S2. Detailed information of primers and RNA sequences used in this study. Table S3. The differentially expressed protein-coding genes in EJ and T24T cell lines upon circPTPRA overexpression. Table S4. The differentially immunoprecipitated transcripts that interacted with IGF2BP1 in T24T cells upon ectopic expression of circPTPRA. [file 12943_2021_1359_MOESM2_ESM.zip › Table. S2.pdf]

Supplementary table 2: The sequences of primers, oligonucleotides and probes used in this study.	
	(5'-3')
IGF2BP1-F	TGAAGCTGGAGACCCACATA
IGF2BP1-R	GGGTCTGGTCTCTTGGTACT
GAPDH-F	AATCCCATCACCATCTTCCAG
GAPDH-R	AAATGAGCCCCAGCCTTC
circPTPRA-F	ATGCCAGAACAGAACCCTGG
circPTPRA-R	ATATCAGACCACTGCCGAGC
circSMARCA5-F	CAAGATGGGCGAAAGTTCA
circSMARCA5-R	GCACCTCTTTCCAAAATACCA
MYC-F	TTCGGGTAGTGGAAAACCAG
MYC-R	AGTAGAAATACGGCTGCACC
FSCN1-F	AAAGTGTGCCTTCCGTACC
FSCN1-R	CCACTCGATGTCAAAGTAGCAG
PTPRA-F	AGTGGTCTGATATGTGTCAGTGC
PTPRA-R	GGTTCTGCCGTTGATGAGTTA
**FISH probes**	
circPTPRA (cy3)	Ribobio
U6 (cy3)	Ribobio
18s (cy3)	Ribobio
**Biotinylated probes**	
Sense circPTPRA probe	AACTTTCCCTCCTTCAGATAAGCATGGATTCCTG
Anti-sense circPTPRA probe	CAGGAATCCATGCTTATCTGAAGGAGGGAAAGTT
**siRNAs**	
si-circPTPRA#1	TCCTTCAGATAAGCATGGATT dTdT
si-circPTPRA#2	CCTCCTTCAGATAAGCATGGA dTdT
Si-NC	AGUACAGCAAACGAUACGGTT dTdT
