# Supplementary figures and images for: Correction: CircPTPRA blocks the recognition of RNA N6-methyladenosine through interacting with IGF2BP1 to suppress bladder cancer progression
Source: Mol Cancer. 2022 Nov 1;21:205. doi: 10.1186/s12943-022-01677-8 (PMC9624052; doi:10.1186/s12943-022-01677-8)

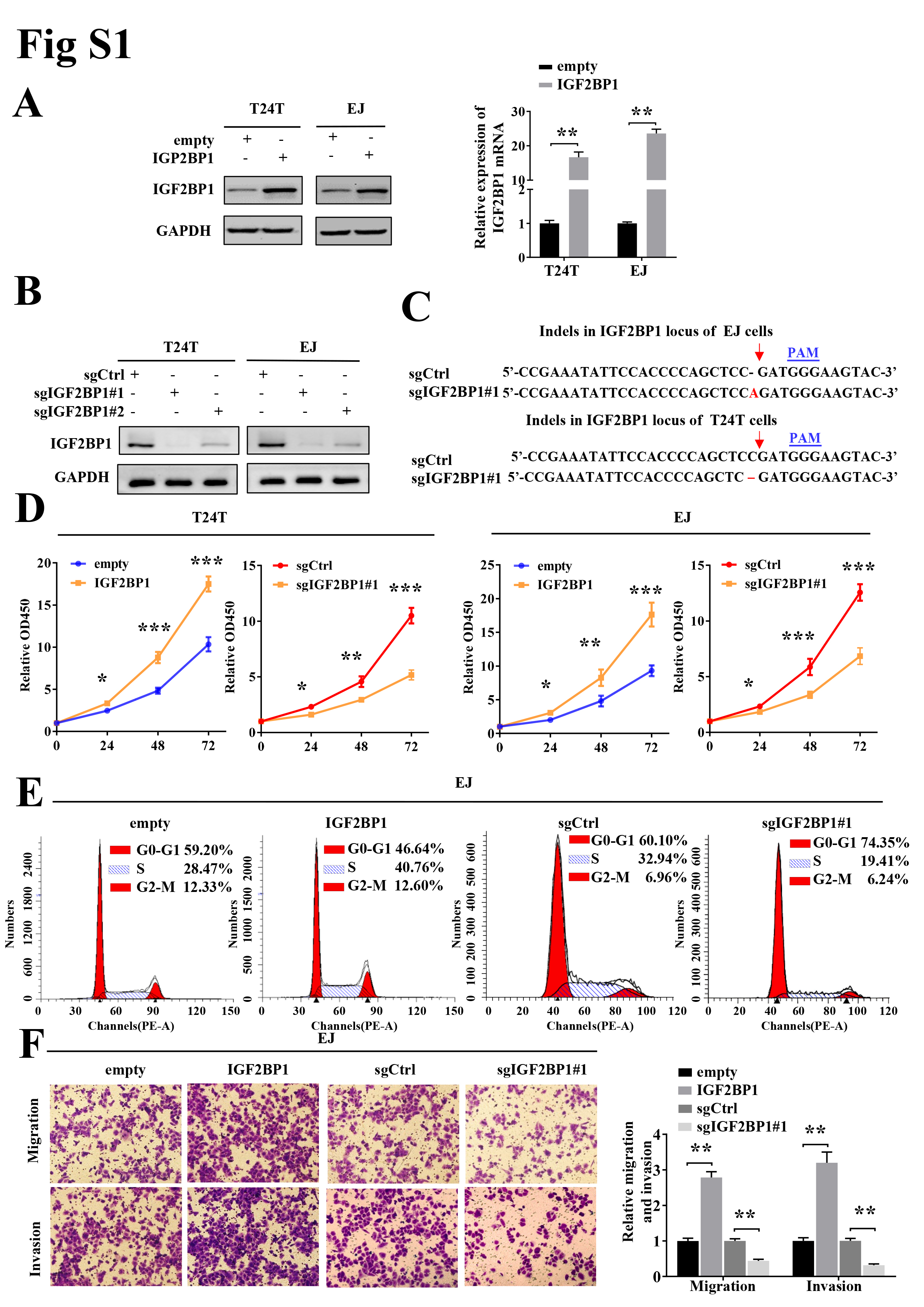

Supplement: Supplementary file 1 — Additional file 1: Figure S1. A. and B. The efficiencies of stable models of IGF2BP1 overexpression or knockout in EJ and T24T cell lines. C. Sanger sequencing of genomic DNAs to validate IGF2BP1 knockout in EJ and T24T cells. The mutation patterns on the two alleles are highlighted in red. The red arrow represents the cleavage site. D. CCK-8 assay was performed to evaluate cell viability in BC cells with stable overexpression or knockout of IGF2BP1. E. and F. Cell cycle analysis and representative images and quantification of transwell assay indicating the proliferation and invasion of EJ cells with stable overexpression or knockout of IGF2BP1. The data are the means ± SEM of three independent experiments. **, P < 0.01. [file 12943_2022_1677_MOESM1_ESM.jpg]
